# Lenvatinib-Associated Vaginal Necrosis and Rectovaginal Fistula After Brachytherapy

**DOI:** 10.7759/cureus.83541

**Published:** 2025-05-05

**Authors:** Keiichiro Baba, Taisuke Sumiya, Takashi Saito, Motohiro Murakami, Hideyuki Sakurai

**Affiliations:** 1 Radiation Oncology, University of Tsukuba Hospital, Tsukuba, JPN

**Keywords:** brachytherapy, endometrial cancers, lenvatinib, radiotherapy (rt), rectovaginal fistula, vaginal necrosis

## Abstract

Lenvatinib and radiotherapy, including brachytherapy, are treatment options for patients with endometrial cancer. However, it is unclear if lenvatinib can induce ulceration and necrosis in patients who have previously received brachytherapy. Here, we describe the case of a 63-year-old woman with endometrial cancer who developed postoperative vaginal wall recurrence and received brachytherapy. One year later, she was given lenvatinib, and two months later, she developed radiation-induced vaginal necrosis, which rapidly progressed to rectovaginal fistula. This case shows that physicians should be aware of the possibility of vaginal necrosis in the administration of lenvatinib to patients who have previously undergone radiotherapy including brachytherapy for the uterus or vagina.

## Introduction

External beam radiotherapy (EBRT) and brachytherapy are used as primary treatment for patients with unresectable endometrial cancer and as salvage treatment for local recurrence. Lenvatinib may also be used as a second-line treatment for endometrial cancer. The combination of lenvatinib and pembrolizumab (LEN + PEM) significantly prolongs progression-free and overall survival compared to conventional chemotherapy in patients with advanced endometrial cancer who have received platinum-based chemotherapy [1]. Based on this study, LEN + PEM is a treatment option for patients with mismatch repair-proficient endometrial cancer after the use of platinum-based chemotherapy.

Lenvatinib is known to cause skin ulcers, gastrointestinal ulcers, and perforation [2-4], while brachytherapy and EBRT for gynecological cancers rarely cause vaginal necrosis and rectovaginal fistula [5]. The risk of gastrointestinal perforation is increased when EBRT is used in combination with vascular endothelial growth factor (VEGF) inhibitors other than lenvatinib [6], but there have been no reports of vaginal necrosis or rectovaginal fistula induced by brachytherapy being exacerbated by the addition of lenvatinib. Here, we report a case of a patient with endometrial cancer who had received EBRT and brachytherapy and subsequently developed vaginal necrosis and a rectovaginal fistula that were suspected to have been caused by lenvatinib.

## Case presentation

**Figure 1:**
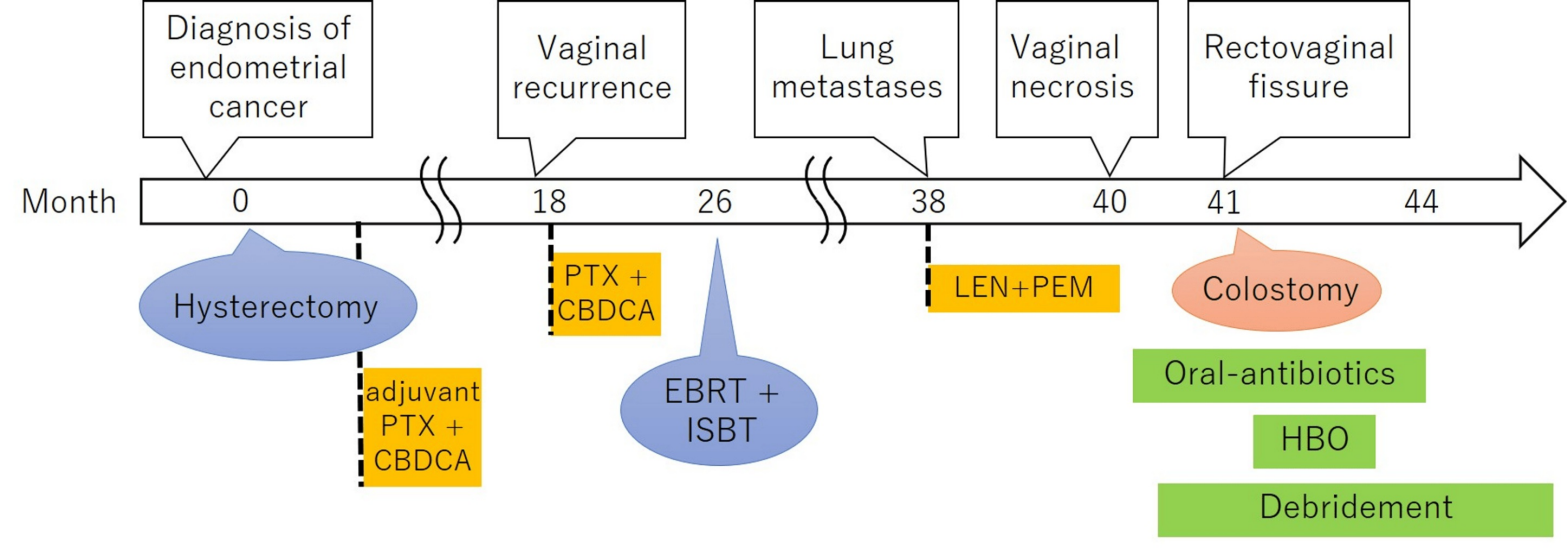
Timeline of the clinical course PTX: paclitaxel; CBDCA: carboplatin; EBRT: external beam radiotherapy; ISBT: interstitial brachytherapy; LEN: lenvatinib; PEM: pembrolizumab; HBO: hyperbaric oxygen therapy

The patient was a 63-year-old woman with no history of smoking or diabetes. She was diagnosed with endometrial cancer after experiencing abnormal genital bleeding and had undergone total abdominal hysterectomy and bilateral salpingo-oophorectomy, pelvic lymphadenectomy, and para-aortic lymphadenectomy. The pathological diagnosis was grade 2 endometrioid carcinoma, pT1bN0M0 Stage IB (TNM Classification 8th edition by Union for International Cancer Control), with 34/40 mm myometrial invasion. She received six courses of paclitaxel and carboplatin as adjuvant chemotherapy after surgery (Figure 1).

**Figure 2:**
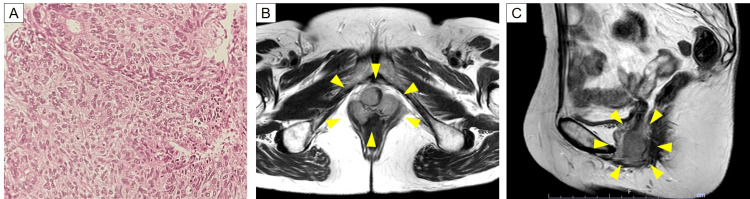
Appearance of vaginal wall recurrence of the endometrial cancer (A) A hematoxylin and eosin-stained image of a nodule on the vagina. The pathological diagnosis was endometrioid carcinoma. (B and C) Magnetic resonance imaging T2-weighted images. The yellow arrowheads indicate the site of the vaginal wall recurrence.

**Figure 3:**
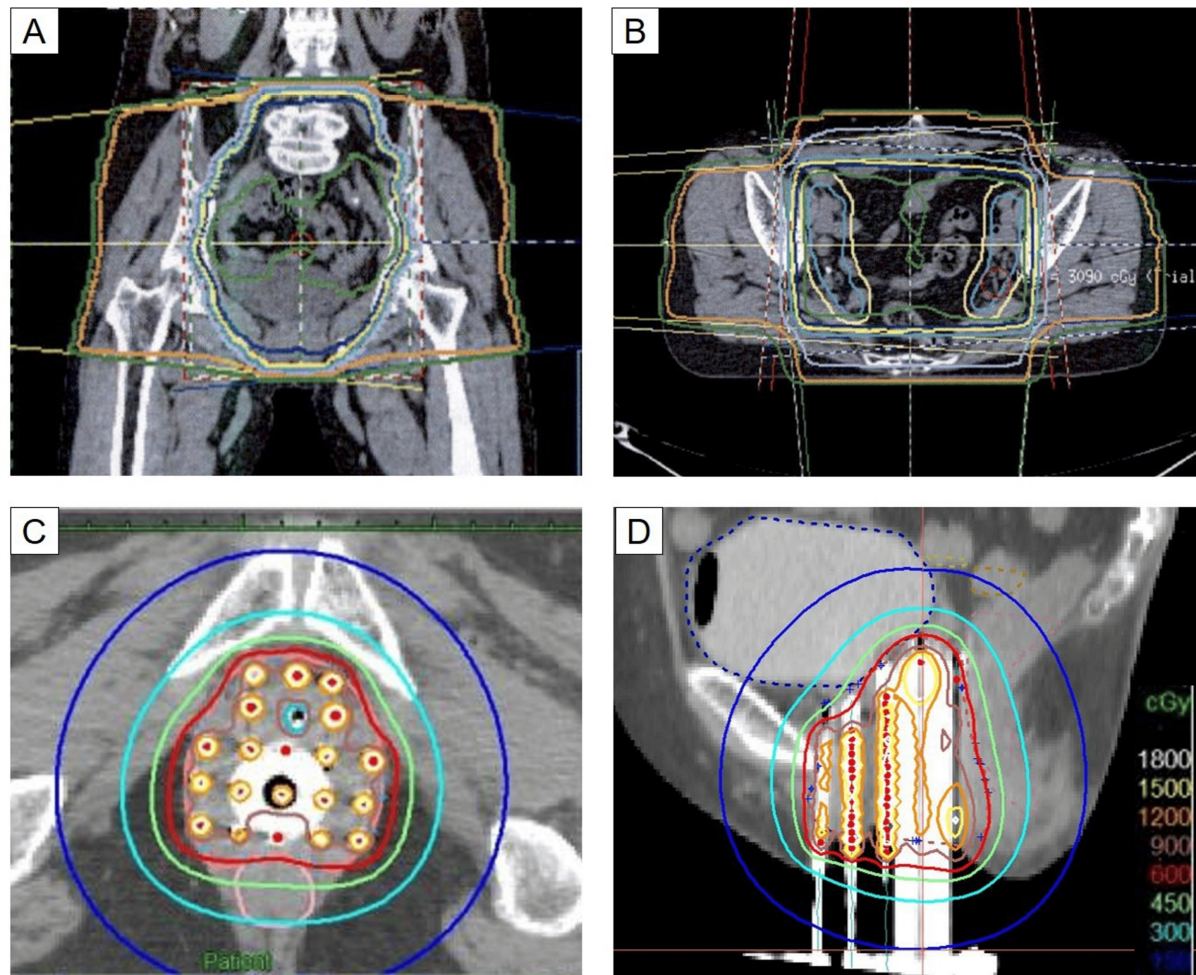
The dose distribution maps for the radiotherapy (A and B) Whole pelvis irradiation. (C and D) Interstitial brachytherapy.

One year and six months after surgery, a nodule appeared at the vaginal entrance and a biopsy was performed. The pathological diagnosis was endometrioid carcinoma, and she was diagnosed with vaginal wall recurrence (Figure 2A). 18F-fluorodeoxyglucose positron emission tomography did not show any metastases outside the vagina. Six courses of paclitaxel plus carboplatin were given, but a computed tomography (CT) scan four months later showed stable disease. Magnetic resonance imaging revealed that the tumor measured 35 mm craniocaudally, 30 mm anteroposteriorly, and 41 mm transversely. The boundaries between the tumor and the rectum and bladder were clearly defined, with no evidence of invasion into either organ (Figures 2B, 2C). The recurrence was treated with EBRT and brachytherapy. EBRT was performed using three-dimensional conformal radiotherapy with a total dose of 50 Gy in 25 fractions, consisting of 30 Gy in 15 fractions to the whole pelvis including the vaginal entrance and 20 Gy in 10 fractions with central shielding. Interstitial brachytherapy was performed using metal needles and a vaginal cylinder, using a high dose rate afterloading system with iridium-192 at 30 Gy in five fractions in three days. Based on the dose constraints of the EMBRACE-II study, we evaluated the summed dose of EBRT and brachytherapy to clinical target volume (CTV) D90 and organs at risk D_2cm_^3^ [7]. Calculating biologically equieffective doses in 2 Gy per fraction (EQD2) with α/β = 3 Gy using the linear quadratic model, the dose limits for organs were set as follows: bladder < 90 Gy, rectum, sigmoid, and bowel < 75 Gy. In the actual treatment, the summed EQD2 to the CTV was 75.7 Gy and 71.7 Gy to the bladder, 72.0 Gy to the rectum, 35.3 Gy to the sigmoid colon, and 35.0 Gy to the bowel. The dose distribution of radiotherapy is shown in Figure 3. After radiotherapy, the recurrence achieved a complete response.

**Figure 4:**
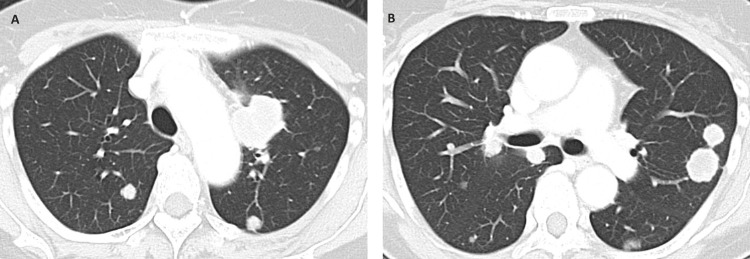
CT scan images of the chest one year after radiotherapy There were multiple nodules in both (A and B) lungs, and the diagnosis was multiple pulmonary metastases.

One year after radiotherapy, a routine CT scan revealed multiple pulmonary nodules in both lungs, leading to the diagnosis of pulmonary metastases from endometrioid carcinoma (Figure 4), and there were no recurrences in the area of the vaginal wall that had previously received radiotherapy. A hysterectomy specimen was tested for microsatellite instability (MSI) and tumor mutation burden (TMB) using OncoGuide NCC Oncopanel System™ (Sysmex Corporation, Kobe, Japan). The MSI score was 42.88 (>30), and the somatic mutation rate was 21.7 mut/Mb (>10). The case was diagnosed as MSI-high and TMB-high.

**Figure 5:**
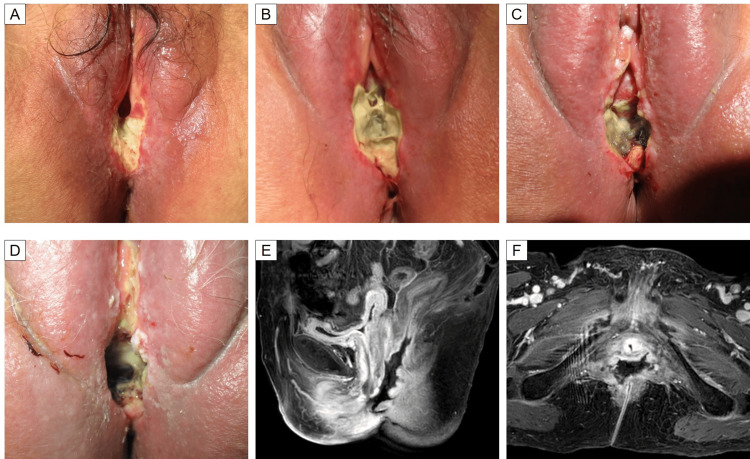
Clinical course of vaginal necrosis (A-D) Photos on days 1, 14, 28, and 61, respectively, from the day the vaginal ulcer was discovered. The ulcer progressed and the entire vagina and part of the urethra became necrotic, with the development of cellulitis on the surrounding skin. (E, F) Gd-enhanced T1-weighted magnetic resonance imaging on day 71. The posterior wall of the vagina and the anterior wall of the rectum have completely disappeared, forming a rectovaginal fistula.

**Figure 6:**
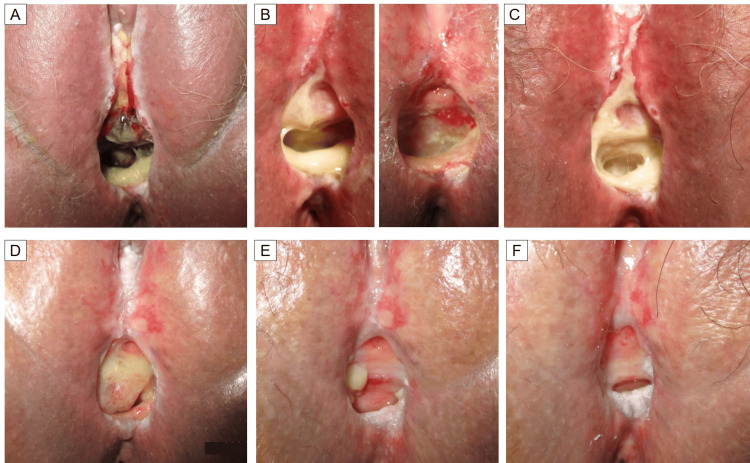
The process of vaginal necrosis healing (A) Day 88 after the detection of vaginal necrosis. (B) Day 144 after the detection of vaginal necrosis. The left image shows pre-debridement, and the right image shows post-debridement. (C) Day 157 after the detection of vaginal necrosis. Two weeks post-debridement from the time in (B), necrotic tissue had again adhered circumferentially within the vaginal cavity. (D-F) Days 235, 347, and 476, respectively, after the detection of vaginal necrosis.

The patient was treated with four courses of oral LEN 20 mg/day and intravenous PEM 200 mg every three weeks. One month after starting LEN + PEM, the patient developed perineal pain, and on examination two months after starting LEN + PEM, an ulcer was found on the posterior wall and both lateral walls of the vagina. A biopsy was taken from the vaginal wall, and the pathological diagnosis revealed only necrotic fibrous connective tissue with no tumor cells detected. Therefore, the lesion was considered not to be a recurrence of endometrial cancer. Over two weeks, the ulcer became necrotic, and cellulitis of the labia majora and perineum developed. LEN + PEM was discontinued two weeks after the discovery of the vaginal ulcer after a total of three courses of administration. After LEN + PEM was discontinued, oral antibiotics were started, with daily washing of the ulcer using an aqueous solution of polysorbate-20 and debridement of necrotic tissue. However, the area of necrosis continued to expand, and a rectovaginal fistula developed one month after the discovery of the ulcer. The course of vaginal necrosis is shown in Figure 5. A transverse colostomy was performed, and the patient then received hyperbaric oxygen therapy (HBO, 100% oxygen, two atmospheres absolute for one hour, 30 sessions/six weeks) and continued oral and topical antibiotics, daily perineal washing, and regular debridement of necrotic tissue. During the debridement process, nasal forceps and iris scissors were employed to excise as much necrotic tissue from the vaginal cavity as possible (Figure 6B).

The perineal cellulitis healed within three months of starting the antibiotics. Subsequently, the area with attached necrotic tissue gradually decreased, and 15 months after the detection of necrosis, all of the skin around the vagina had re-epithelialized and the vaginal necrosis had healed. The process of vaginal necrosis healing is shown in Figure 6. After the completion of HBO therapy, the patient was treated with doxorubicin plus cisplatin for pulmonary metastases. Due to increased pulmonary metastasis, the patient enrolled in a clinical trial with tislelizumab. Following further progression of the pulmonary metastases, the regimen was switched to medroxyprogesterone acetate, and treatment is ongoing.

## Discussion

Lenvatinib is an oral multikinase inhibitor that targets VEGF receptors 1-3, fibroblast growth factor receptors 1-4, platelet-derived growth factor receptor-α, RET, and KIT. The mechanisms through which lenvatinib causes ulceration/perforation include suppression of angiogenesis, reduction of vascular density, arterial thromboembolism, reduction of the mucosal barrier, and epithelial cell apoptosis. In the SELECT study, a phase III trial for thyroid cancer, gastrointestinal perforation or fistula was observed in 2% of patients receiving lenvatinib compared to 0.8% in the placebo group [4].

Radiation-induced vaginal necrosis occurs in 3%-6% of patients who have undergone radical radiotherapy for gynecological cancer [5]. High doses of radiation cause thrombosis and vascular luminal occlusion in microvessels. Chronic ischemia due to this vascular dysfunction, combined with the depletion of basal progenitor cells, significantly reduces wound healing capacity, and external factors such as trauma and infection can cause mucosal necrosis [8]. A review suggested several risk factors for radiation-induced vaginal necrosis, including high total dose, high dose rate, reirradiation, smoking, irradiation of the distal vagina, history of abdominal surgery, and diabetes [5,8]. Radiation-induced vaginal necrosis is often detected as a small white change in the vaginal mucosa, and this initial stage of necrosis progresses to form ulceration and complete necrosis [8].

Radiation-induced rectovaginal fistulae are even rarer than vaginal necrosis and are caused by higher doses of radiation. A retrospective analysis of 36 cases performed to identify risk factors for gastrointestinal toxicity after radical radiotherapy combined with interstitial brachytherapy in patients with gynecological cancer showed that the risk of grade 2 or higher rectovaginal fistula was significantly greater in patients receiving a summed rectal dose ≥ 76 Gy [9]. The EMBRACE study found similar results, with a significantly higher risk (12.5% vs. 0%-2.7%) of rectal fistula in patients with a summed EQD2 (a/b = 3) of the rectum D_2cm_^3^ of ≥75 Gy compared to those receiving <75 Gy [10]. In our case, the combined dose to the rectum from EBRT and brachytherapy was 72.0 Gy, which was below these cut-off doses for fistula formation.

There have been several reports of ulceration and necrosis in irradiated areas other than the vagina in patients who received lenvatinib after radiotherapy [11,12]. A patient with hepatocellular carcinoma treated with proton beam therapy followed by lenvatinib developed a large skin ulcer at the irradiated site five months after starting lenvatinib [11]. In another report, a patient received radical radiotherapy for prostate cancer and was treated with lenvatinib for hepatocellular carcinoma 15 years later. In the initial radiotherapy, the summed EQD2 (α/β = 3 Gy) for the rectum was 95.6 Gy. Four months after starting lenvatinib, a grade 3 rectal ulcer appeared on the anterior wall of the rectum on the irradiated site, and the patient underwent a colostomy. It was concluded that the rectal ulcer was induced by the combination of the previous radiotherapy and the effects of lenvatinib [12]. As in these previous cases, it is suggested that radiotherapy-induced damage to the vaginal mucosa may have interacted with lenvatinib in this case.

As pembrolizumab was administered concurrently with lenvatinib in our case, the possibility that pembrolizumab may have contributed to the vaginal necrosis must be considered. Pembrolizumab is a PD-1 inhibitor that has shown efficacy in patients with advanced endometrial cancer when used in combination with chemotherapy (paclitaxel and carboplatin) or lenvatinib [1,13]. To the best of our knowledge, there have been no reports suggesting an increased risk of vaginal necrosis or rectovaginal fistula with the use of immune checkpoint inhibitors, including pembrolizumab. Furthermore, clinical trials involving the combined use of immune checkpoint inhibitors and radiotherapy for gynecologic malignancies have not reported the occurrence of vaginal necrosis or rectovaginal fistula [14]. Therefore, it is considered unlikely that pembrolizumab contributed to the vaginal necrosis in this case.

There are common mechanisms between epithelial damage caused by lenvatinib and radiation-induced vaginal necrosis. In our case, there were several risk factors for vaginal necrosis, including a history of hysterectomy, high dose rate brachytherapy, and radiotherapy to the distal part of the vagina. However, the recurrent lesion did not invade the rectum, and the radiation dose to the rectum was lower than that considered likely to cause rectovaginal fistula. As vaginal necrosis occurred two months after lenvatinib administration and rapidly progressed to rectovaginal fistula, it is reasonable to hypothesize that lenvatinib had a synergistic effect on the vulnerable tissue after radiotherapy and induced vaginal necrosis, resulting in a rectovaginal fistula.

There are several treatments for radiation-induced vaginal necrosis, including HBO, debridement of necrotic tissue, administration of antibacterial drugs, sitz baths, and pentoxifylline [5]. Many studies have reported the effectiveness of HBO against radiation damage [15]. The necrotic tissue on the surface of the ulcer becomes a breeding ground for bacteria and inhibits the growth of granulation tissue [16]. As with the treatment of skin ulcers in other parts of the body, debridement is also performed for vaginal necrosis [5]. In addition, it is important to establish an early artificial anus in cases of rectovaginal fistula to prevent infection from worsening [17]. In this case, we were able to cure vaginal necrosis by discontinuing lenvatinib and continuing treatment including HBO and debridement.

## Conclusions

We report the first case of severe vaginal necrosis after lenvatinib administration in a patient who had undergone radiotherapy including brachytherapy. Radiotherapy including brachytherapy and lenvatinib are both effective treatments for endometrial cancer, but more attention should be paid to serious side effects, including vaginal necrosis, when lenvatinib is administered to patients who have undergone radiotherapy.
